# Comment on: Food for Bone: Evidence for a Role for Delta-Tocotrienol in the Physiological Control of Osteoblast Migration. *Int. J. Mol. Sci.* 2020, *21*, 4661

**DOI:** 10.3390/ijms21186674

**Published:** 2020-09-12

**Authors:** Kok-Lun Pang, Kok-Yong Chin

**Affiliations:** Department of Pharmacology, Faculty of Medicine, Universiti Kebangsaan Malaysia, Jalan Yaacob Latif, Bandar Tun Razak, Cheras 56000, Kuala Lumpur, Malaysia; pangkoklun@gmail.com

Dear Editor,

We applaud the innovative work by Casati et al., which explored the effects of delta-tocotrienol (δ-TT) in promoting osteoblast migration [1]. Vitamin E is reported as a nutrient important for maintaining bone health in epidemiological studies [2]. However, the statement “osteoporosis has been correlated with low intake, and serum levels of TTs” is inaccurate because dietary tocotrienol level has not been shown to correlate with bone health, probably due to the absence of a reliable dietary questionnaire that could assess the tocotrienol intake. Nevertheless, there is an abundance of preclinical evidence on the beneficial skeletal effects of tocotrienol. Most in vitro studies focus on the differentiation of osteoblasts, while the animal studies used bone cellular histomorphometry to quantify the bone cells in osteopenic rats treated with tocotrienol [3,4]. The work by Casati et al. is the first that focuses on the influence of tocotrienol on osteoblast migration, which plays an essential role in fracture healing. More accurately, mesenchymal stem cells migrated to the fracture site during fibrovascular phase and callus formation will differentiate into osteoblasts and perform bone formation [5]. A previous study also showed that particles incorporated with annatto tocotrienol rich in δ-TT could enhance callus strength of male rats with long bone fracture fixed with plate and screws [6]. The finding of δ-TT enhances the transcriptional activities of β-catenin also echoes our previous study, which demonstrated that annatto tocotrienol supplementation (60 mg/kg/day for 2 months) increased beta-catenin gene expression in the bone of orchidectomized rats [7]. 

We would like to put forward a few recommendations regarding experiments. Casati et al. used LY294002 to prove the involvement of PI3K in mediating the pro-migratory effects of δ-TT on osteoblasts. LY294002 is a prototype PI3K inhibitor which possesses significant off-target effects by inhibiting several other proteins/enzymes in its working concentration (reviewed in [8,9]). We also wonder why assessment of the efficiency of LY294002 in reducing phospho-Akt level, with or without the δ-TT treatment, was not performed. A similar study treating MC3T3-E1 cells with LY294002 showed that at 20 µM, it suppressed the baseline phospho-Akt level by 50% [10]. In the same study, LY294002 also significantly downregulated the β-catenin expression by 70% in the cells [10], suggesting the involvement of other pathways other than PI3k signalling in suppressing β-catenin. We also noted increased cellular debris in the group treated with LY294002 (Figure 5C). LY294002 is known to induce cell death at the concentration of 5–25 µM [11,12] which might interfere with the MC3T3-E1 cell migration. Therefore, specific and potent PI3k inhibitors like pictilisib or dactolisib may be more suitable in this study.

On the other hand, procaine is a common local anaesthetic agent that block voltage-gated sodium channel (reviewed in [13]). At 1 µM, it inhibits osteo/odontogenesis of mesenchymal stem cells by suppressing nuclear translocation of β-catenin and upregulating glycogen synthase kinase-3β [14]. It also reactivates the expression of Wnt inhibitory factor-1 and reduces β-catenin levels in lung cancer cells at higher concentration (0.2–0.5 mM) [15]. It could inhibit DNA-methyltransferases in colon cancer cells at 0.5 mM [16]. Yet, it is not an established Wnt/β-catenin pathway inhibitor due to its broad spectrum of action. We recommend the authors to assess the effects of procaine on glycogen synthase kinase-3β expression and/or β-catenin phosphorylation or nuclear translocation, with or without the δ-TT. An established Wnt/β-catenin inhibitor (reviewed in [17]) or gene silencing [18] would be more conclusive approaches in determining the role of Wnt/β-catenin in mediating the δ-TT on MC3T3 cells. 

Previously, Casati et al. demonstrated that δ-TT induced MC3T3-E1 cell proliferation upon 2 h of treatment [19]. However, in the current study, a 24 h 10 µg/mL of δ-TT inhibited the MC3T3-E1 cell proliferation by causing G1 arrest (Figure 1). The procedure of cell cycle analysis by employing 0.1% Nonidet (assuming it is nonident P-40 detergent) in permeabilization of live cells is not common compared to 70% ethanol fixation. Treatment of live cells with detergents ruptures the cell membrane and allows the entry of fluorochromes like PI to DNA [20]. We suspect the number of mitotic cells was underestimated because mitotic cells lack nuclear membrane, therefore chromosomes and DNAs could leak out from cells during pipetting or mixing [20]. A higher number of mitotic cells (M phase) might form upon δ-TT treatment, but the chromosomes and DNAs of these cells were lost during the pipetting/transferring. Besides, from our experience, a ModFit LT^TM^ software estimates cell cycle phases better via Gaussian curves modelling estimation, in contrast to typical population gating via Cell Quest software. The coefficient of variation (CV) value also could be calculated via the ModFit LT software to ensure optimal consistency (CV < 6%, better if < 3%) [20]. 

Nevertheless, the findings of Casati et al. are eye-opening and call for testing of other individual vitamin E isomers (including alpha-tocopherol, which is the most ubiquitous in our body) and mixture (mimicking common tocotrienol supplements in the market) in the migration of osteoblasts.
